# Biochemical indicators, cell apoptosis, and metabolomic analyses of the low-temperature stress response and cold tolerance mechanisms in *Litopenaeus vannamei*

**DOI:** 10.1038/s41598-024-65851-2

**Published:** 2024-07-02

**Authors:** Weilin Zhu, Qiangyong Li, Min Peng, Chunling Yang, Xiuli Chen, Pengfei Feng, Qingyun Liu, Bin Zhang, Digang Zeng, Yongzhen Zhao

**Affiliations:** Guangxi Key Laboratory of Aquatic Genetic Breeding and Healthy Aquaculture, Guangxi Academy of Fishery Sciences, Nanning, 530021 China

**Keywords:** *Litopenaeus vannamei*, Metabolomics, Cell apoptosis, Low-temperature stress, Biochemical indicators, Functional genomics, Gene regulation, Animal breeding

## Abstract

The cold tolerance of *Litopenaeus vannamei* is important for breeding in specific areas. To explore the cold tolerance mechanism of *L. vannamei*, this study analyzed biochemical indicators, cell apoptosis, and metabolomic responses in cold-tolerant (Lv-T) and common (Lv-C) *L. vannamei* under low-temperature stress (18 °C and 10 °C). TUNEL analysis showed a significant increase in apoptosis of hepatopancreatic duct cells in *L. vannamei* under low-temperature stress. Biochemical analysis showed that Lv-T had significantly increased levels of superoxide dismutase (SOD) and triglycerides (TG), while alanine aminotransferase (ALT), alkaline phosphatase (ALP), lactate dehydrogenase (LDH-L), and uric acid (UA) levels were significantly decreased compared to Lv-C (p < 0.05). Metabolomic analysis displayed significant increases in metabolites such as LysoPC (P-16:0), 11beta-Hydroxy-3,20-dioxopregn-4-en-21-oic acid, and Pirbuterol, while metabolites such as 4-Hydroxystachydrine, Oxolan-3-one, and 3-Methyldioxyindole were significantly decreased in Lv-T compared to Lv-C. The differentially regulated metabolites were mainly enriched in pathways such as Protein digestion and absorption, Central carbon metabolism in cancer and ABC transporters. Our study indicate that low temperature induces damage to the hepatopancreatic duct of shrimp, thereby affecting its metabolic function. The cold resistance mechanism of Lv-T *L. vannamei* may be due to the enhancement of antioxidant enzymes and lipid metabolism.

## Introduction

*Litopenaeus vannamei*, also known as the Pacific white shrimp or white legged shrimp, is a crucial crustacean species in South America and Asian countries., accounting for 51.7% of the total crustacean aquaculture production in 2020^1^. *L. vannamei* has good environmental adaptability, fast growth rate, and high nutritional value, making it economically valuable^2^. However, shrimp lacks the ability to regulate its body temperature and is therefore sensitive to temperature changes^3^. Low temperatures can cause various metabolic disorders in shrimp and even death^4^. Low temperature has an impact on the feeding and swimming speed of *L. vannamei*^5^, and even causes shrimp death when the temperature is below 13 °C^6,7^. Low temperature is a limiting factor for the development of shrimp aquaculture^8,9^, as it restricts the farming seasons and geographical locations, thereby affecting the economic benefits of shrimp farming. Therefore, understanding the cold tolerance mechanism of *L. vannamei* is of great significance for breeding new varieties of cold-tolerant *L. vannamei.*

These physiological responses to environmental changes in organisms often trigger biochemical reactions that affect their metabolism, life cycle, and provide them with physiological adaptability to survive in specific habitats against temperature variations^10–12^. Faced with temperature fluctuations, aquatic animals have a series of response mechanisms to adapt to environmental fluctuations, such as regulating glycolysis and gluconeogenesis, maintaining energy metabolism homeostasis, adjusting membrane fluidity, inducing antioxidant defense systems against reactive oxygen species (ROS), and stimulating immune regulation^13–15^. Studies have shown that the temperature adaptation of *L. vannamei* is closely related to the activity of key enzymes, such as lactate dehydrogenase (LDH), succinate dehydrogenase (SDH), and adenosine triphosphatase (ATPase)^14,16^. Temperature directly affects the metabolism, molting, growth rate, oxygen consumption, survival rate, and indirectly influences shrimp growth through factors like microalgae growth^8^. In addition, temperature significantly affects shrimp hemocyte density^17,18^, as well as antioxidant indicators^19^. After acute cold treatment, the levels of malondialdehyde (MDA) and blood cell DNA damage in shrimp plasma increase significantly^20^. The antioxidant levels in the hepatopancreas of *L. vannamei* significantly increase after acute cold exposure, affecting amino acid, glycerophospholipid, nucleic acid metabolism pathways, and causing damage to the hepatopancreas^14,21^. It has been found that superoxide dismutase (SOD), catalase (CAT), and glutathione peroxidase (GSH-Px) have antioxidant activities and play a role in preventing cell oxidative stress damage during the temperature adaptation of *L. vannamei*^22–24^. Reports have shown that low temperatures can increase dopamine and norepinephrine levels in *L. vannamei*, leading to significant oxidative and antioxidative reactions^25,26^.

However, there are no reports on the metabolic effects of low-temperature stress on cold-tolerant and common shrimp, which hinders a deeper understanding of the molecular mechanisms underlying shrimp's cold tolerance. In our previous study, we successfully bred a cold-tolerant *L. vannamei* strain^27^. In this study, using the cold-tolerant *L. vannamei* as the experimental subject, we investigated blood biochemical parameters, hepatopancreas cell apoptosis, and metabolomic responses of cold-tolerant and common *L. vannamei* under low-temperature stress, aiming to explore the potential molecular mechanisms of cold tolerance in *L. vannamei*.

## Results

### Blood biochemical indices

**Figure 1:**
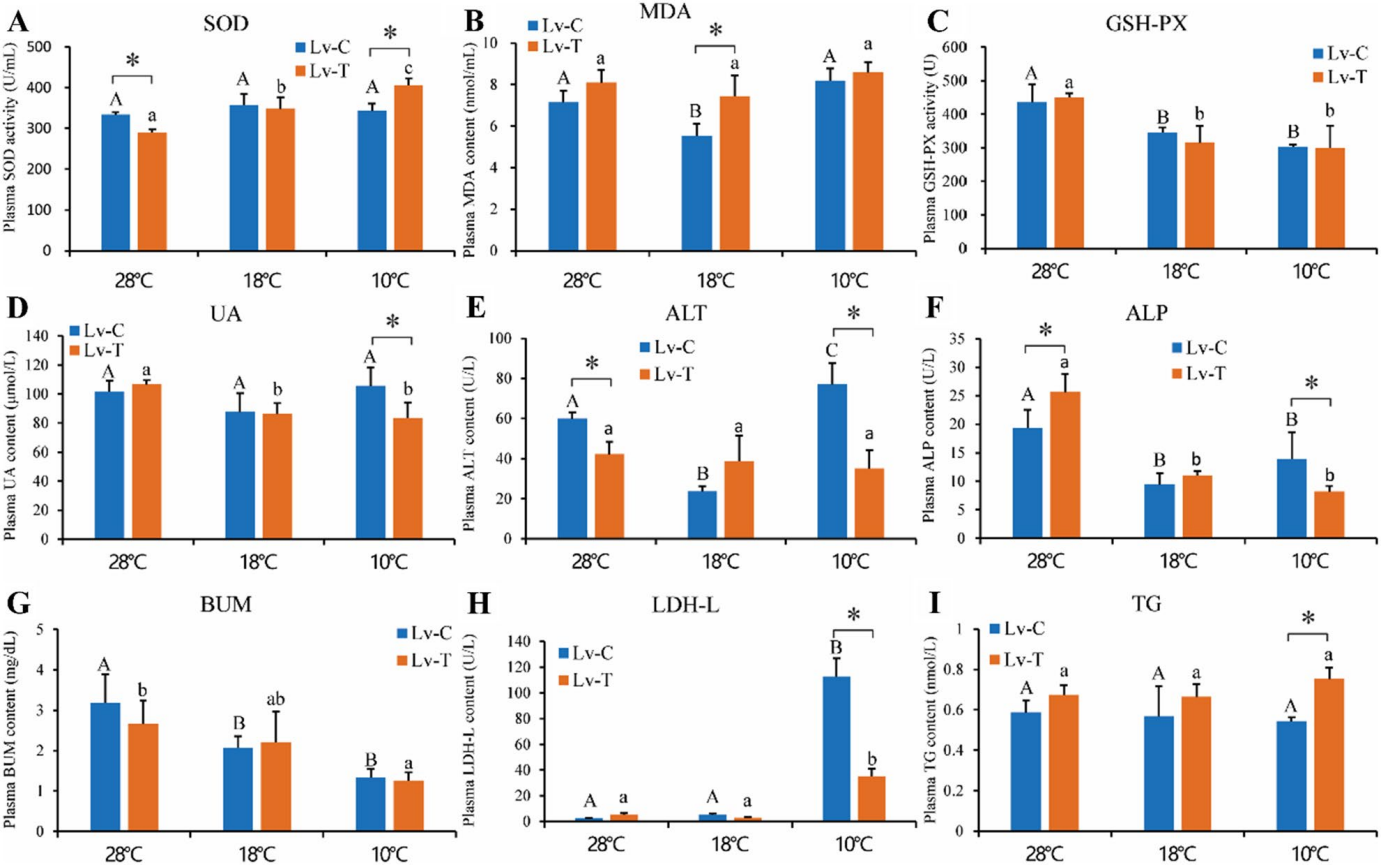
Effects of low temperature stress on blood biochemical indicators of cold tolerant (Lv-T) and common (Lv-C) shrimp. Biochemical indicators include: (**A**) SOD, (**B**) MDA, (**C**) GSH-PX, (**D**) UA, (**E**) ALT, (**F**) ALP, (**G**) BUM, (**H**) LDH-L, and (**I**) TG. The uppercase and lowercase letters represent the differences within the Lv-C and Lv-T groups, respectively. Different letters indicate significant differences (P < 0.05). The difference between two families was compared using t-test.

Under low-temperature stress, both Lv-T and Lv-C shrimp exhibited alterations in indices of antioxidant-related enzymes. The SOD content in the blood of Lv-T shrimp was significantly higher than that of Lv-C shrimp at 10 °C, and it increased significantly as the temperature decreased, while there was no significant change in SOD content in Lv-C shrimp as the temperature decreased (Fig. 1A). Under 18 °C conditions, the MDA of Lv-T shrimp was significantly higher than that of Lv-C shrimp (P < 0.05) (Fig. 1B). GSH-PX content decreased significantly as temperature decreased in both Lv-T and Lv-C shrimp, but there was no significant difference between these two shrimp groups (Fig. 1C). Other enzyme indices also changed under low-temperature stress. TG content in the blood of Lv-T shrimp was significantly higher than that of Lv-C shrimp at 10 °C, while the UA, ALT, LDH-L, and ALP contents were significantly lower in Lv-T shrimp compared to Lv-C shrimp (Fig. 1D–I). Glucose (GLU) and total cholesterol (T-CHO) were also tested, but there were no differences between the different groups (not shown in Fig. 1).

### TUNEL analysis of apoptosis in hepatopancreatic cells of *L. vannamei* under low-temperature stress

**Figure 2:**
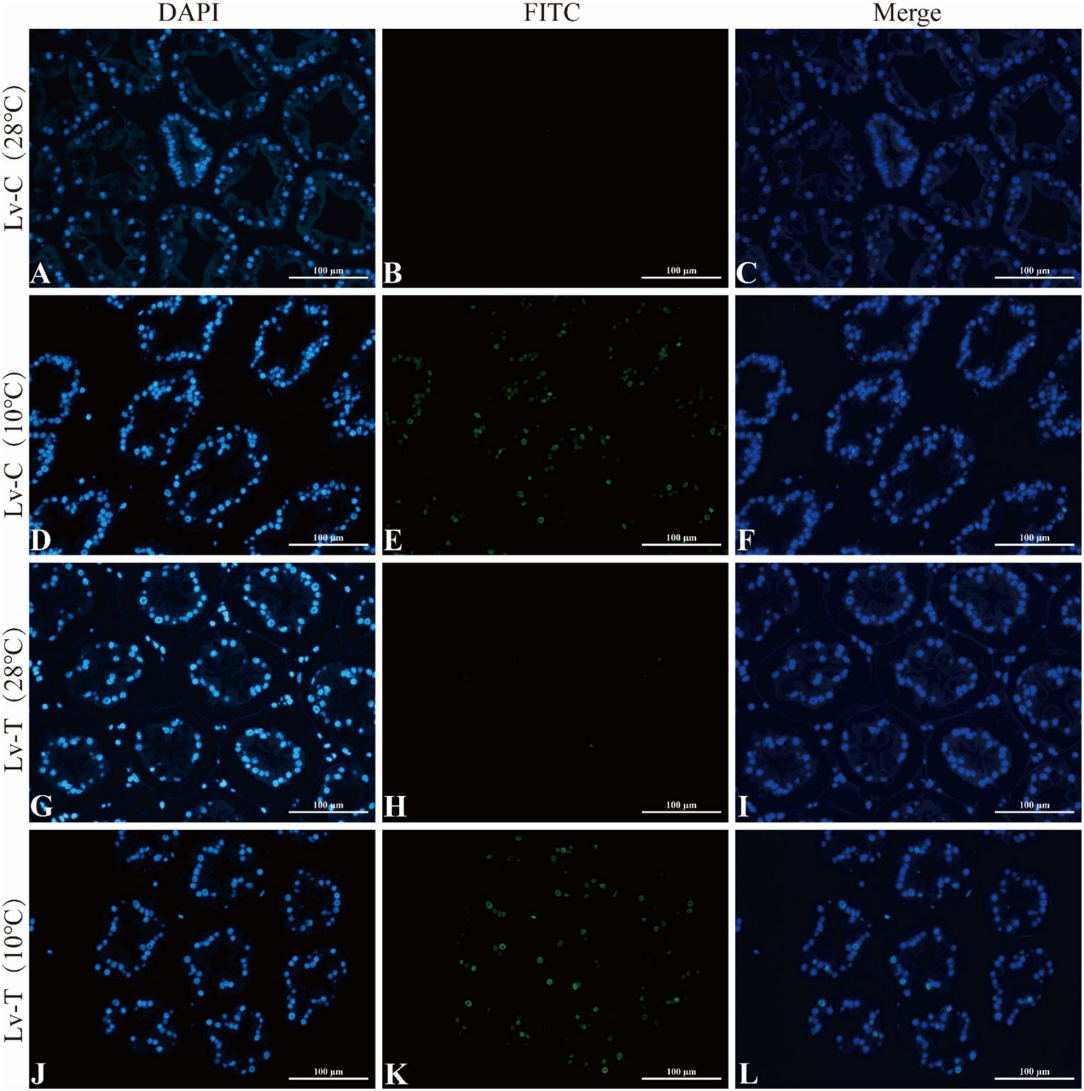
TUNEL assay for hepatopancreatic cells apoptosis in cold tolerant (Lv-T) and common (Lv-C) shrimp under 28 °C and 10 °C. Under UV light, the DAPI-stained nuclei display blue, and the FITC-labeled positive apoptotic nuclei appear green. (**A**) DAPI-stained nuclei in Lv-C shrimp at 28 °C. (**B**) FITC-labeled nuclei in Lv-C shrimp at 28 °C. (**C**) Merge of DAPI-stained and FITC-labeled nuclei in Lv-C shrimp at 28 °C. (**D**) DAPI-stained nuclei in Lv-C shrimp at 10 °C. (**E**) FITC-labeled nuclei in Lv-C shrimp at 10 °C. (**F**) Merge of DAPI-stained and FITC-labeled nuclei in Lv-C shrimp at 10 °C. (**G**) DAPI-stained nuclei in Lv-T shrimp at 28 °C. (**H**) FITC-labeled nuclei in Lv-T shrimp at 28 °C. (**I**) Merge of DAPI-stained and FITC-labeled nuclei in Lv-T shrimp at 28 °C. (**J**) DAPI-stained nuclei in Lv-T shrimp at 10 °C. (**K**) FITC-labeled nuclei in Lv-T shrimp at 10 °C. (**L**) Merge of DAPI-stained and FITC-labeled nuclei in Lv-T shrimp at 10 °C.

To investigate the damage caused by low temperature to the hepatopancreas of *L. vannamei*, we used TUNEL staining to detect apoptosis in hepatopancreatic cells at 28 °C and 10 °C (Fig. 2A–L). Under 28 °C conditions, both Lv-T and Lv-C shrimp showed little or no TUNEL-positive signals in the hepatopancreatic duct wall (Fig. 2B,H). In contrast, strong TUNEL-positive signals were observed on the hepatopancreatic duct walls of both Lv-T and Lv-C shrimp at 10 °C (Fig. 2E,K). The results indicated that cell apoptosis occurred in the hepatopancreatic ducts of both shrimp strains at 10 °C, suggesting that low temperature causes damage to the hepatopancreatic ducts, leading to metabolic disorders.

### Metabolomic analysis

To explore the cold tolerance mechanism of shrimp under low-temperature stress, we performed non-targeted LC–MS/MS metabolomic analysis of the hepatopancreas metabolites of Lv-T and Lv-C shrimp.

**Figure 3:**
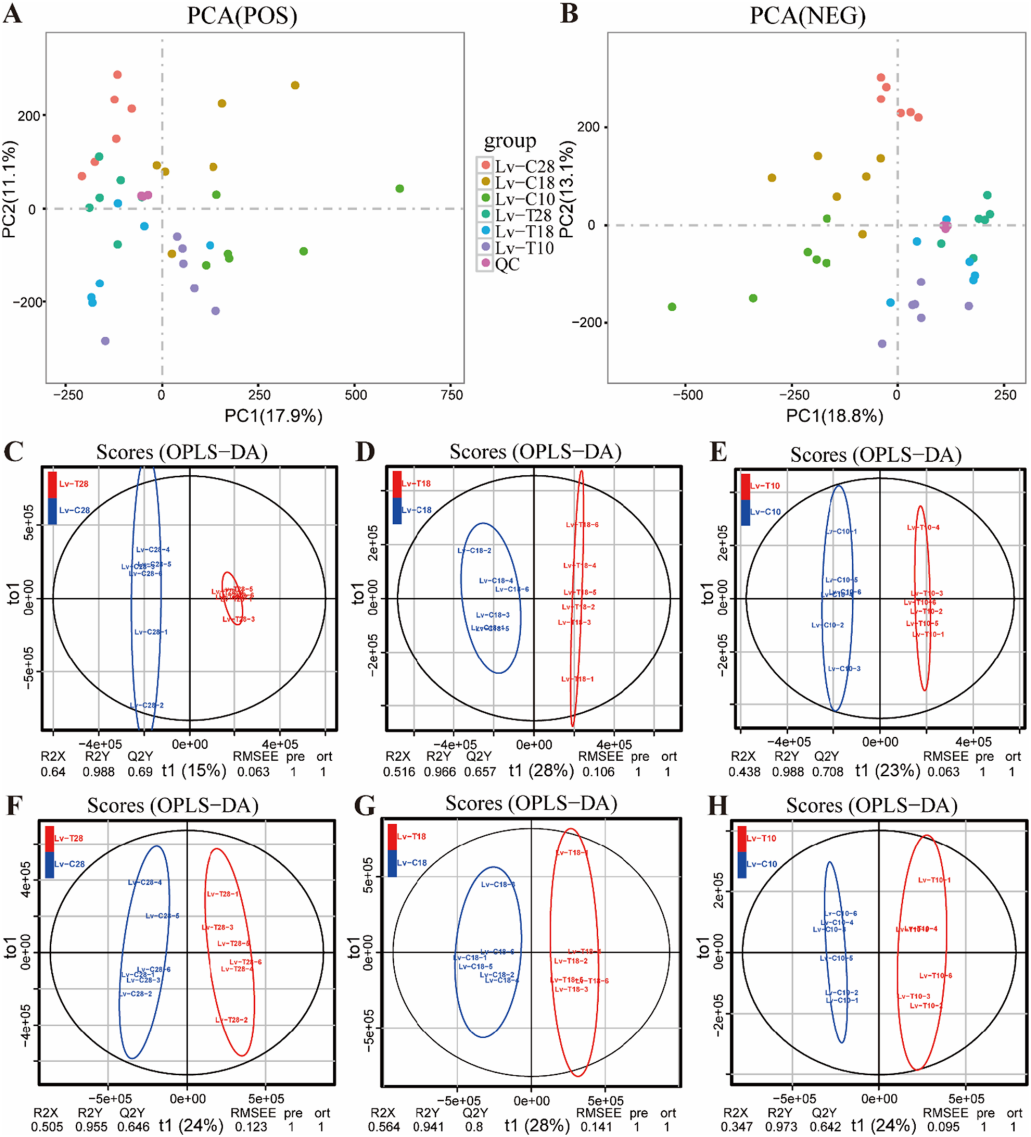
Quality analysis of the metabolomic data. (**A**) and (**B**) represent the Principal Component Analysis (PCA) score plot of samples observed under positive ion mode and negative ion mode, respectively. (**C**–**E**) Represent Orthogonal Partial Least Squares Discriminant Analysis (OPLS-DA) score plots of samples under positive ion mode. (**F**–**H**) represent OPLS-DA score plots of samples under negative ion mode.

A total of 18,176 cationic peak and 14,379 anionic peak were detected by LC–MS/MS detection. A total of 18,176 cationic peak and 14,379 anionic peak were detected by LC–MS/MS. To test the repeatability of the obtained metabolomic data, a multivariate statistical analysis was conducted on the dataset. PCA analysis of all data showed that the clustering of data within each group of samples obtained in positive ion mode and negative ion mode was very close, indicating high reliability and repeatability of the obtained data (Fig. 3A,B). Then, the established OPLS-DA model was subjected to seven-fold cross-validation (Fig. 3C–H), with R2Y = 0.992 cum and Q2 = 0.878 cum for the OPLS-DA score results in positive ion mode, and R2Y = 0.995 cum and Q2 = 0.901 cum for the OPLS-DA score results in negative ion mode. According to the scoring criteria, the model was stable and reliable, and the two groups of samples could be well separated without overlap.

**Figure 4:**
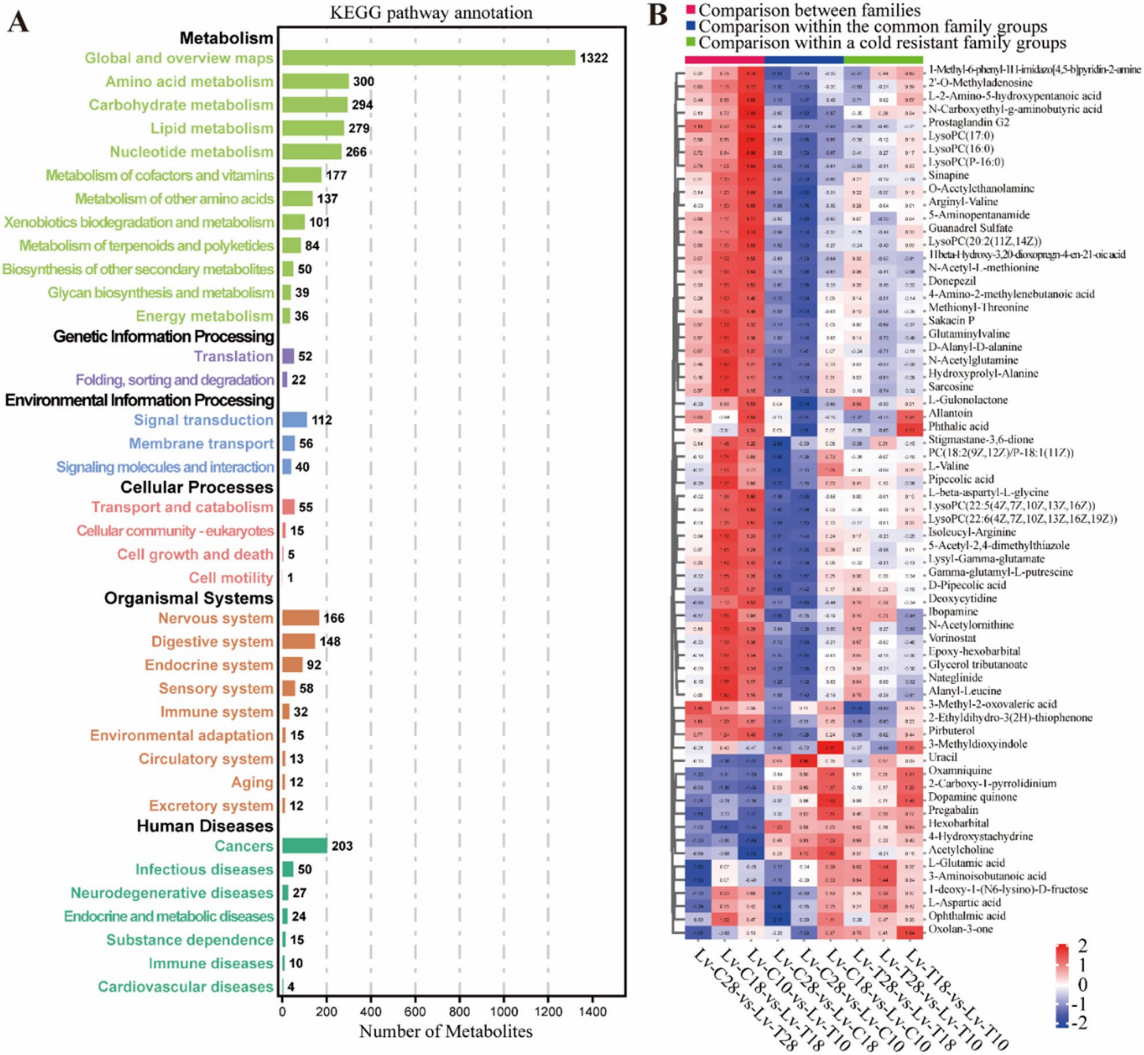
KEGG enrichment analysis of differential metabolites. (**A**) Bar chart showing the number of metabolites enriched in each pathway. (**B**) Heatmap displaying the quantity of significantly different metabolites in various comparison groups, with values represented as Log2 Ratio normalized values. The heatmap was created using the online tools of OmicShare platform (https://www.omicshare.com/tools/home/report/reportheatmap.html) by Guangzhou Genedenovo Biotechnology Co., Ltd.(Guangzhou, China).

We combined OPLS-DA and T-test to screen for significant differential metabolites between different comparison groups. KEGG annotation for differential metabolites revealed that metabolites were predominantly enriched in pathways associated with energy metabolism, such as Global and overview maps, and Lipid metabolism. Furthermore, certain pathways related to Environmental Information Processing, such as Signal transmission and Membrane transport, were also found to be enriched (Fig. 4A).

**Figure 5:**
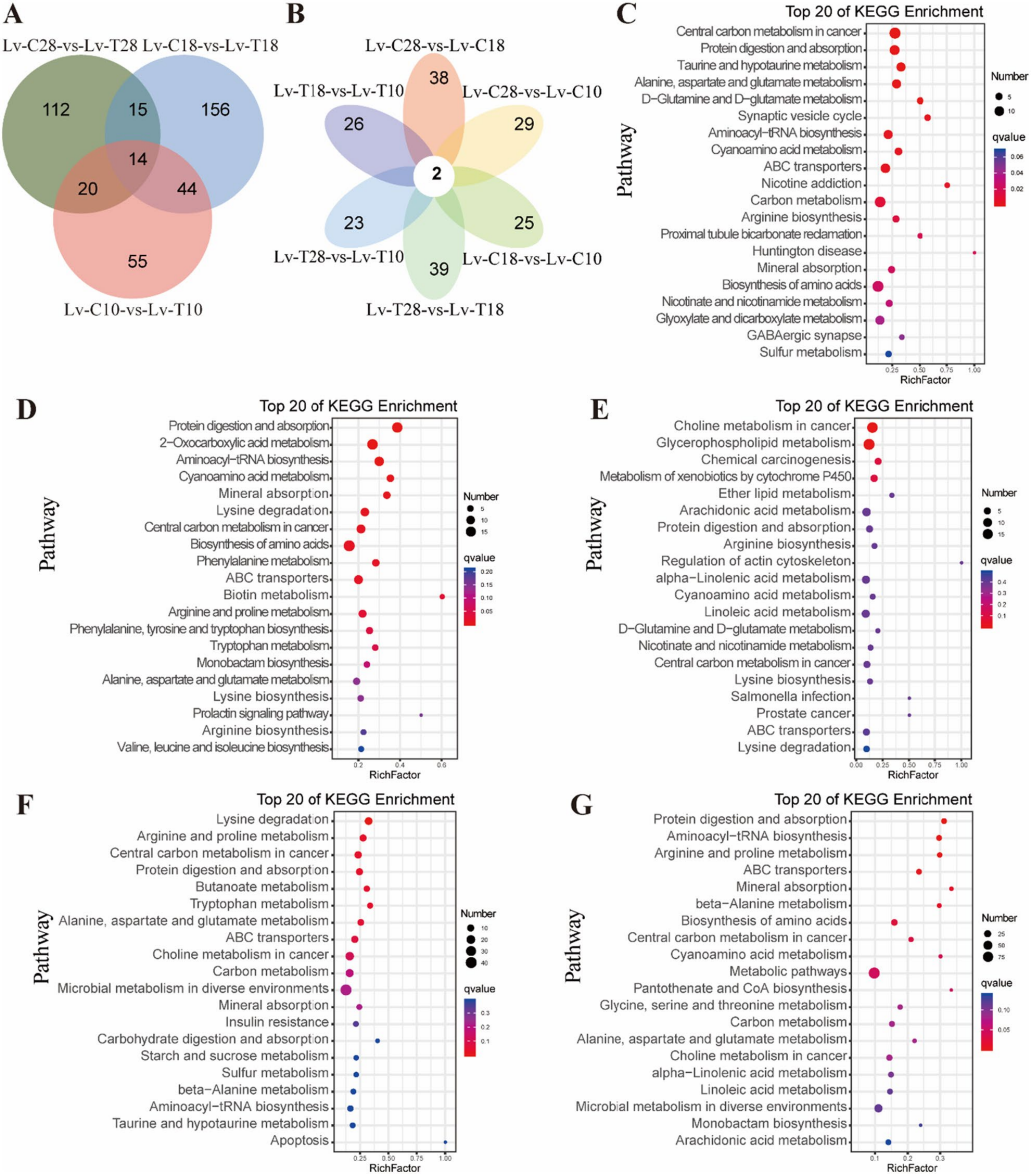
Differential metabolite analysis of hepatopancreas between cold tolerant (Lv-T) and common (Lv-C) shrimp under low-temperature stress. (**A**) The Venn diagram of significant differences in metabolites between Lv-T and Lv-C shrimp under three temperature gradients of 28 °C, 18 °C, and 10 °C. (**B**) Petal plot illustrating the number of significantly different metabolites between different temperature gradients within the same group of shrimp. (**C**) KEGG pathway enrichment of differential metabolites between Lv-T and Lv-C shrimp at 28 °C (Lv-C28-vs-Lv-T28). (**D**) KEGG pathway enrichment of differential metabolites between Lv-T and Lv-C shrimp at 18 °C (Lv-C18-vs-Lv-T18). (**E**) KEGG pathway enrichment of differential metabolites between Lv-T and Lv-C shrimp at 10 °C (Lv-C10-vs-Lv-T10). (**F**) KEGG pathway enrichment of differential metabolites between Lv-C shrimp at 28 °C and Lv-C shrimp at 10 °C (Lv-C28-vs-Lv-C10). (**G**) KEGG pathway enrichment of differential metabolites between Lv-T shrimp at 28 °C and Lv-T shrimp at 10 °C (Lv-T28-vs-Lv-T10).

The total number of substances identified in Lv-T and Lv-C shrimp using the secondary spectrum (MS2) was 416, with 332 cations and 84 anions (Fig. 5A). Comparison between Lv-T and Lv-C shrimp identified 539 MS2 differential metabolites. Common differential metabolites from each comparative group were showed in a heat map (Fig. 4B). Fourteen common metabolites were identified in the comparison between Lv-T and Lv-C shrimp, including the significantly upregulated metabolites of 3-Methyl-2-oxovalic acid (NEG00053), Prostaglandin G2 (NEG00292), and 2'- O-Methyladenosine (POS01044), and significantly downregulated metabolites of Hexobinary (POS00414), Pregabalin (POS00385), and Dopamine quinone (POS00213). In addition, 44 metabolites were common differential metabolites that identitied in low temperature gradient groups, including the upregulated metabolites of LysoPC (P-16:0) (POS00524), 11beta Hydroxy-3,20-dioxoprogn-4-en-21-oic acid (POS01216), and Pirbuterol (POS00152), and the downregulated metabolites of 4-Hydroxystachydrine (POS00073), Oxolan-3-one (POS00312), and 3-Methyldioxyindole (POS00234). Moreover, these metabolites exhibited the same trend changes under low temperature stress within the same shrimp group. *O*-Acetylethanolamine (POS00085) and 5-Aminopentanamide (POS00408) showed significant differences in all comparison groups within the same shrimp group (Fig. 5B). Therefore, we speculate that these are metabolites related to low temperature stress.

We conducted a comparison between Lv-T and Lv-C shrimp, as well as the same shrimp group at different temperature. Compared with Lv-C shrimp, the differential metabolites of Lv-T shrimp were significantly enriched in three temperature gradient comparison groups on the Central carbon metabolism in cancer (ko05230), Protein differentiation and absorption (ko04974), and ABC transporters (ko02010) pathways (Fig. 5C–E). It is worth noting that in the low-temperature response of Lv-T and Lv-C shrimp, there was a significant enrichment of Protein digestion and absorption (ko04974) and ABC transporters (ko02010) (Fig. 5F,G). Additionally, as the temperature decreased, Lysine degradation and Lysine biosynthesis were significantly enriched between Lv-T and Lv-C shrimp at 18 °C and 10 °C. Based on these findings, we hypothesize that these metabolic pathways are closely associated with the response to low-temperature stress.

## Discussion

Among the various environmental factors, water temperature stands out as a critical determinant impacting the physiology of aquatic organisms. Low temperature stress can cause serious damage to the cellular components of aquatic animals, disruption of cellular metabolism, and even death^28,29^. Cold stress leads to high mortality rates in *L. vannamei* and causes significant economic losses^6,7^. Although the physiological, biochemical, transcriptomic, proteomic, and metabolomic responses of *L. vannamei* to temperature fluctuations have been studied^14,21,27,30^, the differential effects of low-temperature stress on the biochemical and metabolic profiles of cold-tolerant and common *L. vannamei* are not well understood.

In the present study, we analyzed the blood biochemistry, hepatopancreatic cell apoptosis, and metabolomic changes in Lv-T and Lv-C *L. vannamei* under low-temperature stress. The analysis of cell apoptosis and biochemical indicators showed that low temperature stress led to apoptosis of cells in the hepatopancreatic duct of *L. vannamei*, increase in blood antioxidant enzyme indicators, and changes in other metabolic related enzyme indicators. Furthermore, the metabolomics analysis uncovered that the impact of low temperature stress on *L. vannamei* was considerable, with a significant alteration observed in the metabolic pathways relating to amino acid metabolism, protein digestion and absorption, glycerol metabolism, and arachidonic acid metabolism.

### Impact of low-temperature stress on physiological and biochemical stability and induction of cell apoptosis

In this study, low-temperature stress affected the biochemical indices of *L. vannamei*. The contents of UA, ALT, LDH-L, and ALP in the blood of Lv-T shrimp were significantly lower than those in Lv-C shrimp at 10 °C (P < 0.05). The decreased temperatures significantly reduced the content of ALP and BUM. These enzyme activities may be related to the adaptive regulation of shrimp^14,16^. Serum alanine aminotransferase (ALT) is a commonly used biomarker for liver health, and increased ALT levels indicate liver disease or injury^31,32^. Previous studies have shown that low temperature leads to increased ALT levels in *L. vannamei*^14^. In this study, the blood ALT content increased in both Lv-T and Lv-C shrimp at 10 °C, but the blood ALT content in Lv-T shrimp was significantly lower than that in Lv-C shrimp, indicating smaller hepatopancreatic damage in Lv-T shrimp. Previous studies have shown that in humans, a decrease in temperature can result in an elevation of UA levels^33^, and UA is considered a marker of purine metabolism impairment^34^. Interestingly, the TG content in the blood of Lv-T shrimp was significantly higher than that in Lv-C shrimp at 10 °C (P < 0.05). The primary role of TG is to supply and store energy while safeguarding the integrity of internal organs^35^. Studies have shown that cold stress in yellow catfish leads to a significant increase in TG consumption^36^. Therefore, the high TG content in Lv-T shrimp at 10 °C may provide energy and protect the hepatopancreas from cold stress.

Under the influence of low temperature stress, there were observable modifications to the levels of SOD, GSH-PX, and MDA in Lv-T and Lv-C shrimp. SOD is the main antioxidant enzyme in organisms. One of the vital functions of SOD in organisms is to mitigate oxidative stress damage by engaging in the removal of ROS^37^. Meng et al.^38^ found that cold acclimation significantly stimulated SOD activity in crab hepatopancreas. Heise et al.^39^ found a significant increase in SOD activity in the liver of the North Sea eelpout (*Zoarces viviparus*) under acute cold stress at 1 °C. GSH-Px eliminates H2O2 using glutathione as a substrate and can also clear lipid peroxides^40^. MDA serves as the end product of lipid peroxidation and serves as an indicator of the extent of oxidative damage incurred by organisms^41^. Moreover, the variations in the activities of antioxidant enzymes are predominantly influenced by variances among species, as well as the intensity and duration of the low temperature stress^28,42,43^. It has been reported that SOD and GSH-Px activities in *L. vannamei* increase significantly under low-temperature stress^14^. In this study, the SOD content in the blood of Lv-T shrimp was significantly higher than that in common shrimp at 10 °C, and it increased significantly as the temperature decreased, while there was no change in SOD content in common shrimp. The GSH-PX indices of both Lv-T and Lv-C shrimp decreased significantly at both 10 °C and 18 °C compared to the temperature groups at 28℃. Based on these results, we speculate that the antioxidant defense system of Lv-T shrimp is effectively activated under low-temperature conditions, and its antioxidant capacity is higher than that of common shrimp, thereby efficiently eliminating reactive oxygen species generated by low temperature.

TUNEL analysis indicated that low temperature caused significant apoptosis in the hepatopancreatic ducts of *L. vannamei*, potentially leading to damage to the hepatopancreas and affecting normal metabolic function. Previous research revealed that acute cold stress caused hepatopancreatic damage in *L. vannamei*, with significant upregulation of genes associated with cell apoptosis and inflammation, such as DRONC, AP-1, and COX-2^44^. Low temperature caused severe morphological damage to the hepatopancreas of *Penaeus japonicus*^45^. The exposure to cold stress exhibited a noteworthy elevation in hepatopancreatic cell apoptosis within red claw crayfish (*Cherax quadricarinatus*)^46^. Wang et al.^19^ reported that acute cold stress can cause hepatopancreatic tissue damage in *L. vannamei* and reduce its immune function. These findings are consistent with the results of this study. Compared to the normal temperature group, both cold-tolerant shrimp and common shrimp exhibited apoptosis at 10 °C. However, the TUNEL results alone did not reveal any differences between the two groups. Therefore, we infer that low temperatures cause damage to the hepatopancreas of both types of shrimp. Low temperatures may lead to the freezing of intracellular water, resulting in frostbite^47,48^. We suggest that even cold-resistant shrimp, when exposed to excessively low temperatures, may experience cell membrane rupture and organelle damage due to water crystallization, ultimately triggering apoptosis. This study found that under the condition of 10 °C, the triglyceride (TG) content in cold-resistant shrimp was significantly higher than that in regular shrimp. The primary role of TG is to supply and store energy while safeguarding the integrity of internal organs^49^. In addition, triglycerides can provide energy for cells to release glycerol and fatty acids, and it is well known that glycerol has a protective effect on cells against frostbite. Therefore, we speculate that TG plays a protective role in the hepatopancreatic cells of cold-resistant shrimp. Previous studies have found that under low-temperature conditions, the cellular redox balance may be disrupted, leading to increased oxidative stress reactions. Oxidative stress can trigger excessive generation of free radicals inside the cells, causing damage to cell membranes, proteins, and nucleic acids, ultimately leading to apoptosis^50–53^. This study found that under the condition of 10 °C, the content of superoxide dismutase (SOD) in cold-resistant shrimp was significantly higher than that in regular shrimp. One of the important functions of SOD in organisms is to alleviate oxidative stress damage by clearing reactive oxygen species (ROS)^54^. Therefore, we speculate that oxidative imbalance occurs in the hepatopancreatic cells of Litopenaeus vannamei after cold stress. However, the SOD levels in cold-resistant shrimp are significantly higher than those in regular shrimp, giving them a greater ability to clear ROS and thus reduce oxidative stress damage. As a result, their survival and adaptation capabilities are enhanced.

### Effects of low-temperature environment on hepatopancreatic metabolism in *L. vannamei*

Metabolomics is considered a powerful tool for detecting fluctuations in metabolites in organisms exposed to environmental stress^55^. LC–MS technology has been widely used in metabolomic analysis of various biological fluids (urine, blood, or tissue extracts)^56^. LC–MS technology has also been extensively applied in metabolomic analysis of *L. vannamei*^57–59^. In our study, we used LC–MS technology to analyze the effects of low temperature on hepatopancreatic metabolism in Lv-T and Lv-C *L. vannamei*. Our metabolomic data showed significant differences in amino acid-related metabolism between Lv-T and Lv-C shrimp under low-temperature stress. Several amino acid metabolites, including N-Acetylornithine, l-2-Amino-5-hydroxypentanoic acid, Lysyl-Gamma-glutamate, Glutaminylvaline, Isoleucyl-Arginine, d-Alanyl-d-alanine, Arginyl-Valine, l-Aspartic acid, *N*-Carboxyethyl-g-aminobutyric acid, l-beta-aspartyl-l-glycine, 3-Methyl-2-oxovaleric acid, Methionyl-Threonine, and Alanyl-Leucine, were significantly more abundant in Lv-T shrimp compared to Lv-C shrimp. Among them, *N*-Acetylornithine is a key intermediate metabolite in the biosynthesis pathway of arginine^60^. The regulation of metabolic pathways for alanine, glutamine, glutamate, arginine, and proline is facilitated through the participation of l-Aspartic acid^61,62^. Arginine participates in hormone secretion and immune regulation^63^. Glutamine is a non-essential amino acid involved in regulating shrimp immunity^64^. Glutamate plays an important role in the physiological response of Drosophila melanogaster to low-temperature domestication^65^. Dopamine quinone is an organic oxygen compound that is neurotoxic to dopamine neuron specific oxidative stress^66^. In our study, the content of dopamine quinone increased in both Lv-T and Lv-C shrimp under low-temperature stress, indicating that low temperature can increase the level of dopamine quinone and cause toxicity to the organism, but the content of dopamine quinone in Lv-T shrimp was lower than that in Lv-C shrimp.

In addition, lipid-related molecules such as LysoPC, Glycerol tributanoate, Sakacin P, Stigmastane-3,6-dione, and 11beta-Hydroxy-3,20-dioxopregn-4-en-21-oic acid were more abundant in Lv-T shrimp compared to Lv-C shrimp, and their levels increased as the temperature decreased. LysoPC plays a regulatory role in immune function^67^, and the LysoPC content in Marsupenaeus japonicus significantly increases under low-temperature stress^45^. Glycerol tributanoate can be hydrolyzed to produce glycerol and butyric acid, which can be metabolized through nutrition and provide energy for small intestinal epithelial cells^68,69^. Glycerol tributanoate can increase the ATP content, activate PIK3 and AKT signal transduction, further activate important Nrf2 signal pathways in cells, enhance the expression of antioxidant enzyme genes, and thus enhance antioxidant enzyme activity^70^. Therefore, the high level of Glycerol tributanoate in Lv-T shrimp may provide energy to resist cold stress and enhance antioxidant capacity. Research has shown that 11beta-Hydroxy-3,20-dioxopregn-4-en-21-oic acid is one of the biomarkers activated by peroxisome proliferator-activated receptor alpha (PPARα). PPARα plays an important role in controlling energy homeostasis and immune regulation^71^.

In this study, we found that under low temperature conditions, cold resistant shrimps had higher levels of glycerophospholipid metabolism, arachidonic acid metabolism, and α- Significant enrichment of alpha linolenic acid metabolism pathway. Previous studies have shown that under low temperature conditions, there are significant differences in the levels of glycerides, phospholipids, sphingolipids, and sterols in the plasma of newborn lambs compared to room temperature conditions^72^. Our results indicate that lipid-related metabolic pathways may be related to the cold tolerance traits of Lv-T shrimp.

## Conclusion

In this study, we conducted blood biochemistry, hepatopancreatic cell apoptosis, and metabolomic analysis of Lv-T and Lv-C *L. vannamei* under low-temperature stress. TUNEL analysis revealed damage to the hepatopancreatic ducts of *L. vannamei* caused by low temperature, which affected their metabolic function. Blood biochemistry analysis showed that the antioxidant enzyme content was significantly higher in Lv-T shrimp compared to Lv-C shrimp. Metabolomic analysis showed that the content of some lipid-related metabolites and antioxidant-related metabolites was significantly higher in Lv-T shrimp compared to Lv-C shrimp. Therefore, the cold tolerance of Lv-T shrimp may be attributed to enhanced antioxidant enzyme activity and lipid metabolism.

## Materials and methods

### Experimental animals and cold stress

Cold-tolerant (Lv-T) and common (Lv-C) *L. vannamei* were obtained from the Guangxi White Shrimp Genetic Breeding Center (Fangchenggang, Guangxi, China). Lv-T exhibited significantly higher survival time and survival rate at 10 °C compared to Lv-C. The performance parameters have been described in our previous study^27^.

Before the experiment, all shrimp were cultured in seawater with a temperature of 28 ± 0.5 °C, salinity of 30‰, and pH of 7.9 ± 0.1 for one month. The average body weight of shrimp was about 32 g. The shrimp were divided into three different temperature groups: 28 °C, 18 °C, and 10 °C. The temperature of 28 °C represented the normal water temperature during the experiment. The temperature of 18 °C and 10 °C were achieved by gradually lowering the water temperature from 28 °C at a rate of 1 °C/h using a cooling system. After reaching the designated temperature, sampling was conducted after 24 h of maintenance. Each group collected 6 biological replicate samples, with each biological replicate composed of a mixture of samples from 6 shrimp. The hepatopancreas samples were preserved in liquid nitrogen. In addition, three replicates were randomly selected from each group, and each replicate consisted of a mixed sample of plasma from six shrimp (1 mL blood extracted from each shrimp with 1/10 sodium citrate anticoagulant) for physiological and biochemical index analysis. Three replicates of hepatopancreas samples were preserved with 4% paraformaldehyde for apoptosis detection.

### Blood biochemical index analysis

The extracted blood samples of *L. vannamei* was centrifuged at 4 °C and 3000 rpm in a centrifuge for 10 min, and the supernatant of each comparison group was collected for biochemical indicators detection. The concentrations of the following enzymes were measured using an enzyme-linked immunosorbent assay (SpectraMax-190, Molecular Devices, USA) and a fully automated biochemical analyzer (3100, Hitachi, Japan). The total superoxide dismutase (SOD) activity, glutathione peroxidase (GSH-PX), and malondialdehyde (MDA) contents in the blood samples were measured using a reagent kit provided by Nanjing Jiancheng Company. The concentrations of alanine aminotransferase (ALT), alkaline phosphatase (ALP), blood urea nitrogen (BUN), uric acid (UA), triglycerides (TG), and lactate dehydrogenase (LDH-L) indicators were measured using a reagent kit provided by Changchun Huili Company. The data analysis was conducted using SPSS 19.0 for statistical analysis. Single factor analysis of variance (ANOVA) was used for comparison between cold resistant and normal families within each group. T-test was used for comparison between the two families, with P < 0.05 indicating significant differences.

### TUNEL analysis for hepatopancreas cell apoptosis

Hepatopancreas tissue sections were prepared. After the fixation was completed, the hepatopancreas samples were slowly flushed with water for 1 h to remove formaldehyde, and were dehydrated with gradient alcohol from low to high in 70% ethanol for 2 h, 80% ethanol for 2 h, 90% ethanol for 1 h, 95% ethanol for 30 min/2 times, and 100% ethanol for 30 min/1 times. The tissue was subjected to two rounds of transparency, each lasting for 15 min. The tissue underwent paraffin embedding four times using a paraffin embedding machine. The embedded tissue was washed four times with melted paraffin and subsequently soaked in dissolved paraffin for 4 times/h. Following the manufacturer's instructions, the fixed tissue samples were sliced into thin sections with a thickness of 4–6 µm using a microtome (Leica-RM2016). After the slicing was completed, the slices were gently placed in 42 °C water and unfolded. Clean glass slides were used to remove the slices from the water, placed in an insulation box and dry at 37 °C, and stored at room temperature for future use. UNEL reagent kit (Roche) was used to perform fluorescence staining on the prepared paraffin sections according to the instructions. The slices were treated with a fixative solution containing anti-fluorescence quenching sealing agent (Servicebio). Finally, the slices were observed under a fluorescence microscope.

### Metabolic profiling analysis of hepatopancreas

The hepatopancreas of *L. vannamei* were used to extract metabolites. The extraction solvent consisted of acetonitrile, methanol, and water in a ratio of 2:2:1, including an internal standard. After vortexing, homogenization, and ultrasound treatment, the samples were incubated at − 20 °C for 1 h. Subsequently, they were centrifuged at 12,000 rpm and 4 °C for 15 min. The resulting supernatant was collected and stored at − 80 °C for future use.

For analysis, the sample underwent LC–MS/MS using an UHPLC system (1290, Agilent Technologies), and MS data was collected using Xcalibur 4.0.27 (Thermo). The MS raw data (.raw) files were converted to mzML format using ProteoWizard and processed using the R package XCMS (version 3.2) (Smith et al., 2006). Samples containing metabolites that were present in less than 50% of all samples in a group were filtered out. Normalization was performed on the filtered samples, resulting in a data matrix containing retention time (RT), mass-to-charge ratio (m/z) values, and peak intensity. The processed data was annotated using OSI-SMMS (version 1.0) software.

Principal Component Analysis (PCA) was conducted on all samples using the R package model (http://www.r-project.org/). The OPLS-DA model was then validated through cross-validation and 200 permutation tests. In each permutation test, class labels were randomly assigned 200 times to generate distributions of R2' and Q2' values. The most distinct metabolites between the two groups were ranked based on the Variable Importance in Projection (VIP) score of the (O)PLS model. Differential metabolites between the two groups were identified using a VIP threshold set at 1, a P-value < 0.05, and a VIP ≥ 1 in the T-test. KEGG enrichment analysis was performed on these differential metabolites^73–75^.

## Data Availability

All non-targeted metabolomic data used in this publication is deposited in the EMBL-EBI MetaboLights database with the identifier MTBLS8493. The complete dataset can be accessed at https://www.ebi.ac.uk/metabolights/MTBLS8493.
